# Does co-inoculation of *Lactuca serriola* with endophytic and arbuscular mycorrhizal fungi improve plant growth in a polluted environment?

**DOI:** 10.1007/s00572-018-0819-y

**Published:** 2018-01-23

**Authors:** Rafał Ważny, Piotr Rozpądek, Roman J. Jędrzejczyk, Marta Śliwa, Anna Stojakowska, Teresa Anielska, Katarzyna Turnau

**Affiliations:** 1 Małopolska Centre of Biotechnology, Jagiellonian University, Gronostajowa 7a, 30-387 Kraków, Poland; 2 Institute of Environmental Sciences, Jagiellonian University, Gronostajowa 7, 30-387 Kraków, Poland; 3 Department of Phytochemistry, Institute of Pharmacology, Polish Academy of Sciences, Smętna 12, 31-343 Kraków, Poland

**Keywords:** *Lactuca serriola*, *Mucor*, *Trichoderma*, Arbuscular mycorrhiza, Endophytic fungi, Co-inoculation

## Abstract

Phytoremediation of polluted sites can be improved by co-inoculation with mycorrhizal and endophytic fungi. In this study, the effects of single- and co-inoculation of *Lactuca serriola* with an arbuscular mycorrhizal (AM) fungus, *Rhizoglomus intraradices*, and endophytic fungi, *Mucor* sp. or *Trichoderma asperellum*, on plant growth, vitality, toxic metal accumulation, sesquiterpene lactone production and flavonoid concentration in the presence of toxic metals were evaluated. Inoculation with the AM fungus increased biomass yield of the plants grown on non-polluted and polluted substrate. Co-inoculation with the AM fungus and *Mucor* sp. resulted in increased biomass yield of plants cultivated on the polluted substrate, whereas co-inoculation with *T. asperellum* and the AM fungus increased plant biomass on the non-polluted substrate. In the presence of *Mucor* sp., mycorrhizal colonization and arbuscule richness were increased in the non-polluted substrate. Co-inoculation with the AM fungus and *Mucor* sp. increased Zn concentration in leaves and roots. The concentration of sesquiterpene lactones in plant leaves was decreased by AM fungus inoculation in both substrates. Despite enhanced host plant costs caused by maintaining symbiosis with numerous microorganisms, interaction of wild lettuce with both mycorrhizal and endophytic fungi was more beneficial than that with a single fungus. The study shows the potential of double inoculation in unfavourable environments, including agricultural areas and toxic metal-polluted areas.

**Electronic supplementary material:**

The online version of this article (10.1007/s00572-018-0819-y) contains supplementary material, which is available to authorized users.

## Introduction

In nature, vegetation is almost always accompanied by fungi and bacteria which often are invisible to observers but can significantly influence plant biology. In degraded environments, the diversity of the plant and soil microbiome usually is severely limited; thus, restoration attempts require utilization of carefully selected microorganisms. The majority of the studies concerning the role of fungi in conferring plant toxic metal (TM) stress tolerance were conducted with arbuscular mycorrhizal fungi (AMF). This group of microorganisms can improve plant growth and adaptation to unfavourable habitats such as industrial wastes, areas surrounding them or those under continuous influence of anthropogenic pressure (Orłowska et al. 2005; Turnau et al. 2010). Their importance in non-polluted environments is just as significant (Jeffries et al. 2003). Although laboratory experiments often confirm the effect of these fungi on plant growth, the results of field studies are not always unequivocal.

Co-inoculation studies, where plants are inoculated by more than one type of microorganism, are a rarity. Investigations including the role of the abiotic environment in such cases are even less common. Descriptions of multi-organismal associations almost exclusively concern co-inoculation with *Rhizobium* and plant growth promoting bacteria (PGPB) (Remans et al. 2008; Ahmad et al. 2011), ectomycorrhizal fungal species and mycorrhizal helper bacteria (MHB) (Frey-Klett et al. 2007) or AMF and bacteria (Liu et al. 2012; Bona et al. 2016). The effect of co-inoculation usually is beneficial for plant growth (Remans et al. 2008; Liu et al. 2012), but Flor-Peregrín et al. (2014), while investigating co-inoculation with AMF and endophytic bacteria, found that co-inoculation had a negative effect on plants compared to single inoculation with AMF or endophytes. Thus, in order to gain a comprehensive understanding of the role of symbiotic microorganisms in plant biology, studies that include different types of microorganisms inhabiting the plant host in multi-microbe setups are necessary. The lack of such studies makes it difficult to understand the complexity of the symbiosis between plants and fungi (Omacini et al. 2006).

The use of mycorrhizal fungi and rhizospheric bacteria in phytoremediation has been reported previously as reviewed by Rozpądek et al. (2017) and Martin et al. (2017). Recently, the fitness of *Verbascum lychnitis* grown in Zn-Pb industrial substrate was shown to be improved by co-inoculation with an arbuscular mycorrhizal (AM) fungus and fungal endophytes (Wężowicz et al. 2017). Endophytes, which colonize plant tissues without causing any negative effects (Hirsch and Braun 1992; Rodriguez et al. 2009), can increase root and shoot biomass (Varma et al. 1999; Omacini et al. 2006; Soleimani et al. 2010) and can protect plants against pathogens and unfavourable environmental conditions such as high temperatures and salinity (Redman et al. 2002; Rodriguez et al. 2008).

*Lactuca serriola* L. (wild lettuce) is a common weed, considered a pioneer of open habitats (Lebeda et al. 2004) because of its high tolerance of poor water and nutrient availability (Gallardo et al. 1996). A unique feature of this species is its ability to orient its leaves in the north-south direction, thereby limiting water loss (Werk and Ehleringer 1985). Wild lettuce is abundantly found along roadsides, abandoned fields, field margins and forest clearings (Weaver and Downs 2003), and interestingly, also on Zn-Pb tailings (Turnau et al. 2012). *L. serriola* recently has been proposed to be used for monitoring soil pollution (Le Guédard et al. 2012). The genus *Lactuca* has been shown to produce characteristic secondary metabolites, sesquiterpene lactones, which accumulate in latex components called laticifers (Michalska et al. 2009). These lactones are the source of the bitterness of wild lettuce leaves and increase their repellence to herbivores (Rees and Harborne 1985).

The aim of this research was to broaden our view regarding the response of plants associated with multiple microorganisms in a toxic metal enriched environment. We investigated the interaction between *L. serriola* and an arbuscular mycorrhizal (AM) fungus and fungal endophytes that were isolated from plants growing on industrial wastes in Southern Poland. Recently, Rozpądek et al. (2018) have shown the importance of a *Mucor* strain for plant fitness and metal homeostasis. This fungus is an endophyte that colonizes both the roots and the shoots of *Arabidopsis arenosa*. The strain was selected for use in the current research in order to verify its potential to colonize and exert its beneficial effect on species other than *A. arenosa*. *L. serriola*, being a mycorrhizal plant, was inoculated with *Mucor* sp. and the effect was compared to another endophytic fungus, *Trichoderma asperellum* Samuels, Lieckf. & Nirenberg, of known behaviour and growth-improving potential (Viterbo et al. 2010).

## Methods

### Plant, fungi and substrate

Seeds of *L. serriola* (collected from plants in the vicinity of Kraków, Poland) were surface sterilized in 8% sodium hypochlorite for 5 min, followed by 96% ethanol for 1 min and 75% ethanol for 3 min and washed five times with sterile deionized water and then germinated in sterile conditions on Murashige and Skooq (MS) medium diluted four times and with added sucrose. The germination was conducted at 4 °C in darkness for 2 days, followed by 14 h photoperiod at 21/17 °C. Two weeks later, seedlings were transferred into MS medium and, after 2 days of adaptation, they were inoculated with the endophytic fungi *Mucor* sp. (NCBI accession number KU234656; strain UNIJAG.PL.50 from *Arabidopsis arenosa* (L.) Hayek seeds) or *Trichoderma asperellum* (NCBI accession number MG571529; strain UNIJAG.PL.6 from *Deschampsia cespitosa* (L.) P.B. leaves). Five days after inoculation, the plants were transferred to pot cultures with polluted (P) or non-polluted (NP) substrate and with or without AM fungus inoculum.

The NP substrate was a mixture of garden soil (supplied by ARO, Poland; pH 5–6.5; N-NO_3_, 100–300 mg/L; P, 80–300 mg/L; K, 150–450 mg/L), sand and clay in equal volumes. The P substrate was made by adding an additional volume of the substrate collected from the industrial waste site, Trzebionka (Poland 50° 09′ 34.5″ N, 19° 25′ 17.2″ E) (Orłowska et al. 2005), to the ARO soil sand and clay mixture (1:1:1:1; *v*/*v*/*v*/*v*). Both substrates were supplemented with 100 g/L rock phosphate (Siarkopol, Poland). Available P (Colwell 1963), Kjeldahl N and organic matter concentrations in the substrates were measured according to Wilke (2005). Zn, Cd, Pb, Fe and K concentrations in the substrates also were investigated. The water content in samples (at 105 °C) was determined by a moisture analyser, and then the samples were digested in 65% nitric acid (5 ml) for 2 h (room temperature—1 h, at boiling point—1 h). After cooling, 1.65 cm^3^ of 30% H_2_O_2_ was added and the suspension was heated to the boiling point. The suspension was centrifuged for 15 min at 3000 rpm, and the supernatant was transferred to a graduated flask. The precipitate (if observed) was treated with deionized water and shaken until a suspension formed anew; this suspension was centrifuged. The supernatant was transferred to a graduated flask containing the solution after the first centrifugation. This procedure was repeated five times. The precipitate was dried and the possible metal content was evaluated by X-ray fluorescence spectroscopy. The solution in the flask was made up to 25 cm^3^ with deionized water. This method involves acid digestion that dissolves all the elements present in the material (Huguet et al. 2015). To determine metal concentrations, atomic absorption spectrometry (flame atomic absorption spectrometry [FAAS] or graphite furnace atomic absorption spectrometry [GF-AAS], equipped with Zeeman Effect background correction and a CSX 260 auto-sampler [Thermo Scientific, iC 3000]) was used. Detailed characteristics of the substrates are shown in Table 1.

**Table 1:** Chemical properties of the non-polluted (NP) and polluted (P) substrates used in the experiment. Different letters within each column indicate statistically significant differences at *P* ≤ 0.05 (*n* = 3) by *t* test

	pH (in KCl)	P_available_ (mg/kg)	K (g/kg)	Organic matter (%)	N (%)	Zn (mg/kg)	Cd (mg/kg)	Pb (mg/kg)	Fe (g/kg)
NP	6.6 ± 0.1 a	19.3 ± 4.1 b	2.5 ± 0.3 a	6.4 ± 0.1 a	0.3 ± 0.04 a	52.0 ± 2.1 b	7.2 ± 1.9 b	0.4 ± 0.1 b	2.5 ± 0.4 b
P	7.3 ± 0.2 a	34.7 ± 10.2 a	2.6 ± 0.1 a	6.0 ± 0.1 a	0.1 ± 0.05 a	917.3 ± 21.9 a	33.1 ± 2.0 a	6.3 ± 0.1 a	6.3 ± 0.2 a

Data present mean ± standard error

Mycorrhizal inoculum was prepared in pot cultures of *Plantago lanceolata* L. with *Rhizoglomus intraradices* (N.C. Schenck & G.S. Sm.) Sieverd., G.A. Silva & Oehl. Approximately 5 ml of the inoculum, containing spores, mycelium and colonized root fragments was mixed with the upper layer of the experiment substrates. Only sand with clay (AM fungus inoculum carrier) was added to control plants. The experiment was a fully crossed, three factor design with main factors of substrate (NP and P) × AM fungus (+ and −) × endophyte (none, *Mucor* sp. or *T. asperellum*) performed in 3 replicate Sunbags × 5 pots/Sunbag (i.e., 180 plants in 36 Sunbags; Sigma-Aldrich, USA) in a greenhouse at 22 °C in natural light for 9 weeks from March to May. Each plant (one per pot) was provided with 0.6 L of substrate and irrigated once a week with 8 ml long Ashton nutrient solution (0.08 M KNO_3_; 0.008 μM (NH_4_)_6_Mo_7_O_24_·4H_2_O; 0.01 M Ca(NO_3_)_2_).

### Chlorophyll and flavonoid measurement

Chlorophyll *a* and flavonoid concentrations were measured in two leaves (without removing them) of each 8-week-old plant (*N* = 15/treatment) with a Dualex Scientific fluorometer (Force-A, France) according to the manufacturers’ instructions. This instrument enables non-destructive assessment of leaf Chlorophyll *a* and flavonoid indices from light transmission.

### Fluorescence of chlorophyll *a* and the JIP test

Chlorophyll fluorescence measurements were performed with a Handy Pea fluorimeter (Hansatech Instruments, UK). Before the measurement, two mature leaves, without removing them, of each plant (9 weeks old) were dark-adapted for 20 min in a special clips. Data were processed with the BIOLYZER software (Laboratory of Bioenergetics, Geneva, Switzerland). Each fluorescent transient was calculated according to the JIP test (Tsimilli-Michael and Strasser 2008). The following multi-parametric indices were used to assess plant vitality (Strasser et al. 2000):

PI_ABS_ is the performance of the photosynthesis apparatus expressed in relation to absorption:

$$ {\mathrm{P}\mathrm{I}}_{\mathrm{ABS}}=\frac{\mathrm{RC}}{\mathrm{ABS}}\times \frac{\upvarphi_{\mathrm{P}0}}{1-{\upvarphi}_{\mathrm{P}0}}\times \frac{\uppsi_0}{1-{\uppsi}_0} $$

where $\frac{\mathrm{RC}}{\mathrm{ABS}}$ is a measure of the fraction of reaction centre chlorophyll (Chl_RC_) per chlorophyll of the antennae (Chl_Antenna_). $\frac{\upvarphi_{\mathrm{P}0}}{1-{\upvarphi}_{\mathrm{P}0}}$ indicates the contribution of light reactions for primary photochemistry according to the JIP test. Electron transport beyond *Q*_a_ (primary quinone acceptor) is quantified as $\frac{\uppsi_0}{1-{\uppsi}_0}$.

Performance index (PI_total_):

$$ {\mathrm{PI}}_{\mathrm{total}}={\mathrm{PI}}_{\mathrm{ABS}}\times \frac{\mathrm{RE}}{\mathrm{ABS}} $$

where $\frac{\mathrm{RE}}{\mathrm{ABS}}$ indicates the contribution of the reduction of end equivalents.

### Plant biomass

Nine-week-old plants were collected and evaluated for the fresh and dry weight. For dry weight measurement, plants were air dried at room temperature for 2 weeks. Dry weight was used for determination of mycorrhizal dependency (MD), calculated according to the Plenchette et al. (1983) index:

$$ \mathrm{MD}=\frac{{\mathrm{DW}}_{\mathrm{M}}-{\mathrm{DW}}_{\mathrm{NM}}}{{\mathrm{DW}}_{\mathrm{M}}}\times 100\ \left(\%\right) $$

where DW_M_—dry weight of mycorrhizal plants; DW_NM_—dry weight of non-mycorrhizal plants. This mycorrhiza dependency formula considers the plant response to mycorrhizas. Plants with a mycorrhizal dependency close to 100% are considered as fully dependent on AM.

### Fungal colonization

Endophytic colonisation in plant tissues stained according to Atsatt and Whiteside (2014) was observed with light field microscopy (Olympus BX53). For the estimation of mycorrhizal colonization, roots were prepared according to the modified Phillips and Hayman (1970) method. The roots were washed in tap water, cleared in 10% of KOH for 24 h, washed again, acidified in 5% lactic acid for 2 h and stained in 0.01% aniline blue in pure lactic acid for 24 h at room temperature. Stained roots were stored in pure lactic acid, cut into 1 cm pieces and mounted in glycerol on microscopic slides. At least 45 root pieces per plant were analysed. Mycorrhizal frequency (F%), absolute mycorrhizal colonization (m%) and absolute arbuscular richness (a%) were assessed (Trouvelot et al. 1986; http://www2.dijon.inra.fr/mychintec/Mycocalc-prg/download.html).

### Toxic metal concentrations

Zn, Cd and Pb concentrations in leaves and roots were measured according to the method described for the substrates (“Plant, fungi and substrate” subsection).

### Sesquiterpene lactone content

Dry, pulverized plant shoots (0.1 g) were treated twice with 10 ml of CH_3_OH at room temperature. The pooled extracts were evaporated in vacuo and the residue was dissolved in 70% CH_3_CN (1 ml), left to stand overnight at 4 °C, centrifuged (11.340×*g*, 5 min) and analyzed by RP-HPLC/DAD method according to Stojakowska et al. (2012). Samples (5 μl) were injected into a Purospher RP-18e (3 × 125 mm, particle size 5 μm) column (Merck, Darmstadt, Germany) and eluted with a mobile phase consisting of water and CH_3_CN, at a flow rate of 1 ml min^−1^, at 40 °C. Gradient elution conditions described by Grass et al. (2006) were applied. Typical retention times of the analyzed sesquiterpene lactones were as follows: lactucin (LC)—9.3 min, lactucopicrin (LCPikr) —30.8 min and 8-deoxylactucin (8-DeoxyLC)—25.5 min. Quantification was performed by measurement of peak areas at 260 nm with guaianolide crepidiaside A as the reference.

### Statistics

Statistical comparisons were performed using Statistica 12.5 (StatSoft) and were considered significant at *P* ≤ 0.05. Data normal distribution and variance homogeneity were assessed with Shapiro-Wilk’s and Levene’s tests, respectively. If necessary, data (chlorophyll *a* fluorescence) were normalized with a log10 transformation. Differences were tested by two-way (mycorrhizal colonization, toxic metal concentration) and three-way (chlorophyll and flavonoid index, chlorophyll *a* fluorescence, plant fresh and dry biomass, lactones concentrations) analysis of variance (ANOVA) followed by the Tukey’s post-hoc test (Supplementary Table 1). Non-polluted and polluted substrates were compared by *t* test. This test was also applied for comparison of JIP test components between tested treatments and the control treatment.

## Results

### Chlorophyll *a* and flavonoids

**Fig. 1:**
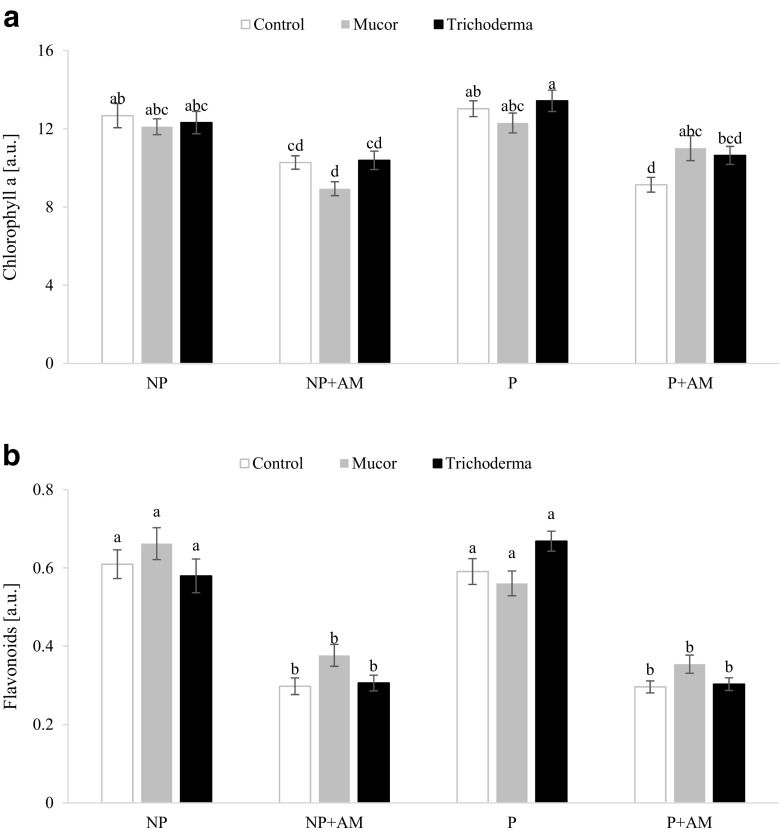
Chlorophyll *a* (**a**) and flavonoid (**b**) concentrations in the leaves of 8-week-old *Lactuca serriola* plants inoculated with the AM fungus (*Rhizoglomus intraradices*) and endophytic fungi (*Mucor sp*. or *Trichoderma asperellum*) and cultivated in non-polluted (NP) and polluted (P) substrate. Bars topped by the same letter do not differ significantly at *P* ≤ 0.05 by Tukey’s test; error bars show standard error; a.u. arbitrary unit

AM fungus inoculation decreased Chlorophyll *a* concentration in plants grown on NP and P substrates (Fig. 1a). Endophytic *Mucor* increased chlorophyll *a* concentration when co-inoculated with AM fungus on P, whereas *T. asperellum* did not affect it. Total flavonoid concentration was significantly higher in plants grown without AM fungus inoculum (Fig. 1b). Endophytic fungi *Mucor* and *T. asperellum* had no effect on total flavonoid concentration.

### Plant vitality

**Fig. 2:**
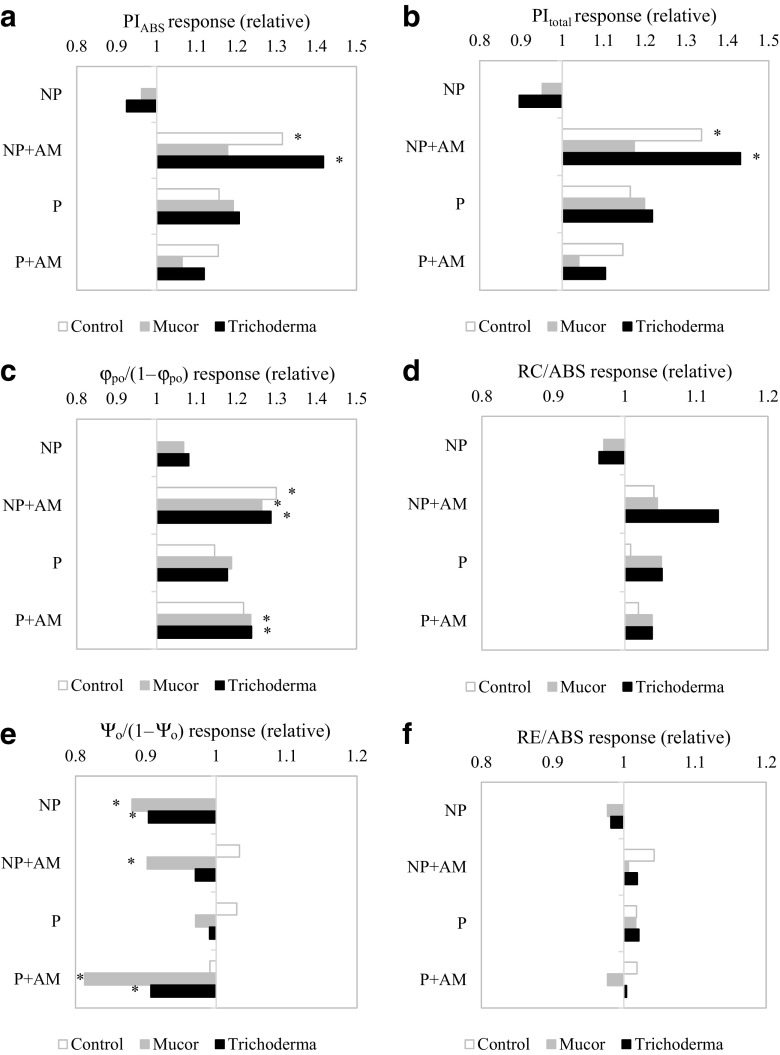
PSII efficiency of plants inoculated with AM (*Rhizoglomus intraradices*) and endophytic (*Mucor* sp. or *Trichoderma asperellum*) fungi compared to non-inoculated plants cultivated on non-polluted (NP) and polluted (P) substrates. JIP test parameters: PI_abs_—absorbance performance index (**a**), PI_total_—total performace index (**b**), φ_P0_/(1 − φ_P0_)—contribution of light reactions for primary photochemistry (**c**), RC/ABS—fraction of reaction centre chlorophyll per chlorophyll of the antennae (**d**), Ψ_0_/(1 − Ψ_0_)—electron transport beyond primary quinone acceptor (**e**) and RE/ABS—contribution of the reduction of end equivalents (**f**) are presented relative to entirely non-inoculated plants; statistically significant differences between particular treatments and those entirely non-inoculated plants are indicated by asterisk (*t* test, *P* ≤ 0.05)

In NP soil, PI_ABS_ and PI_total_ were significantly higher in AM fungus inoculated and AM fungus/*Trichoderma* co-inoculated plants in comparison to non-inoculated plants (Fig. 2a, b). For AM fungus and *Mucor* co-inoculated plants, a similar trend was observed but statistically significant differences were not found. The contribution of light reactions for primary photochemistry $\frac{\varphi_{\mathrm{P}0}}{\left(1-{\varphi}_{\mathrm{P}0}\right)}$ was significantly higher for all of the AM fungus treatments grown in the NP soil (Fig. 2c). Electron transport beyond primary quinone acceptor (*Q*_a_) significantly decreased by endophyte and AM fungus/*Mucor* co-inoculated plants in the NP substrate (Fig. 2e). PI_ABS_ and PI_total_ were not significantly increased by AM fungus- and/or endophyte-inoculation of the plants cultivated in the P substrate nor were the fraction of reaction centre chlorophyll per chlorophyll of the antennae (RC/ABS) and the contribution of the reduction of end equivalents (RE/ABS; Fig. 2a, b, d, f). Co-inoculation enhanced the contribution of the light reactions for primary photochemistry (Fig. 2c) and decreased electron transport beyond *Q*_a_ in comparison to non-inoculated plants in the P substrate (Fig. 2e).

### Plant biomass

**Fig. 3:**
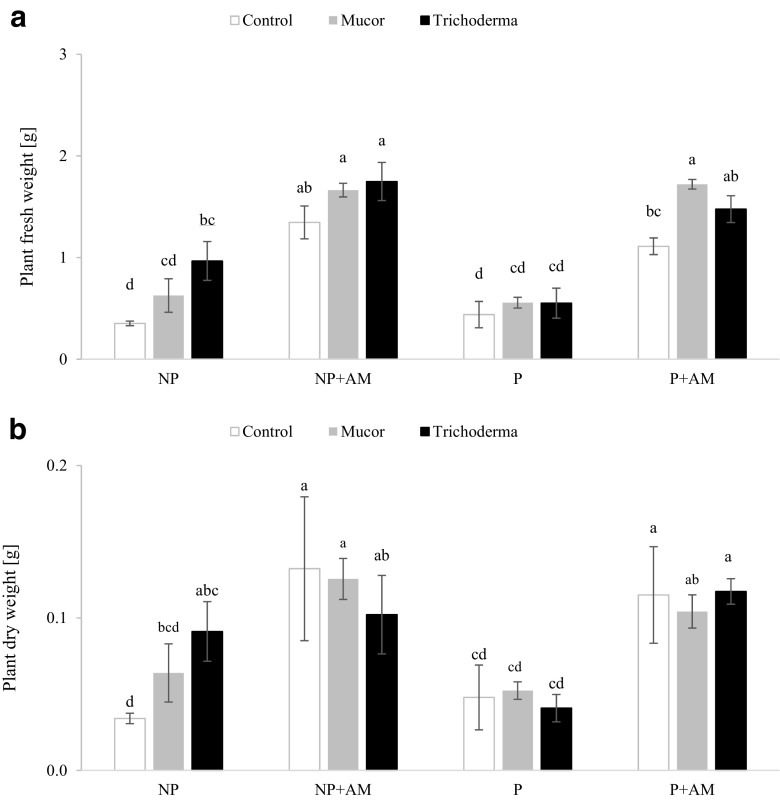
Fresh (**a**) and dry (**b**) weight of *Lactuca serriola* plants inoculated with AM (*Rhizoglomus intraradices*) and endophytic (*Mucor sp*. or *Trichoderma asperellum*) fungi and cultivated on non-polluted (NP) and polluted (P) substrates. Bars topped by the same letter do not differ significantly at *P* ≤ 0.05 by Tukey’s test; error bars show standard error

Inoculation with the AM fungus significantly increased the fresh weight of plants in both the substrates (Fig. 3a; Supplementary Fig. 1). Inoculation with *Mucor* sp. did not affect plant fresh weight. *T. asperellum* increased it on NP, but not on P (Fig. 3a). Co-inoculation with AM fungus and *Mucor* sp. resulted in significantly higher fresh biomass yield of the plants cultivated on P substrate (Fig. 3a). Dry weights of plants grown on NP and P substrates were positively affected by AM fungus-inoculation (Fig. 3b). *Trichoderma* inoculation increased plant dry weight on NP, but co-inoculation did not affect this parameter (Fig. 3b).

The mycorrhizal dependency (MD) index of *L. serriola* inoculated with *Mucor* sp. and *T. asperellum* grown on the NP soil reached 50 and 10%, respectively, and was lower than for plants not inoculated with endophytes (70%). On P substrate, MD of *Mucor*-inoculated plants was similar to non-inoculated plants (51%) and *T. asperellum* increased plant dependency on mycorrhiza to 67%.

### Fungal colonization

Endophytic fungi were easily visible in a few day old seedlings cultivated in vitro. They colonized plant roots either through root hairs (mostly near the tips of root hairs, where the cell wall is thinnest) or close to the meristematic, apical region of young roots, close to the area where abundant root hairs were formed. (Supplementary Fig. 2a, c). The mycelium also was visible when branch roots were formed. *Mucor* hyphae were found mainly in association with root hairs (Supplementary Fig. 2b). *T. asperellum* developed visible mycelium on the root surface (Supplementary Fig. 2d). In older roots of plants cultivated in pots, both fungi were visible growing between cortical cells and causing irregularity of plant cells.

**Fig. 4:**
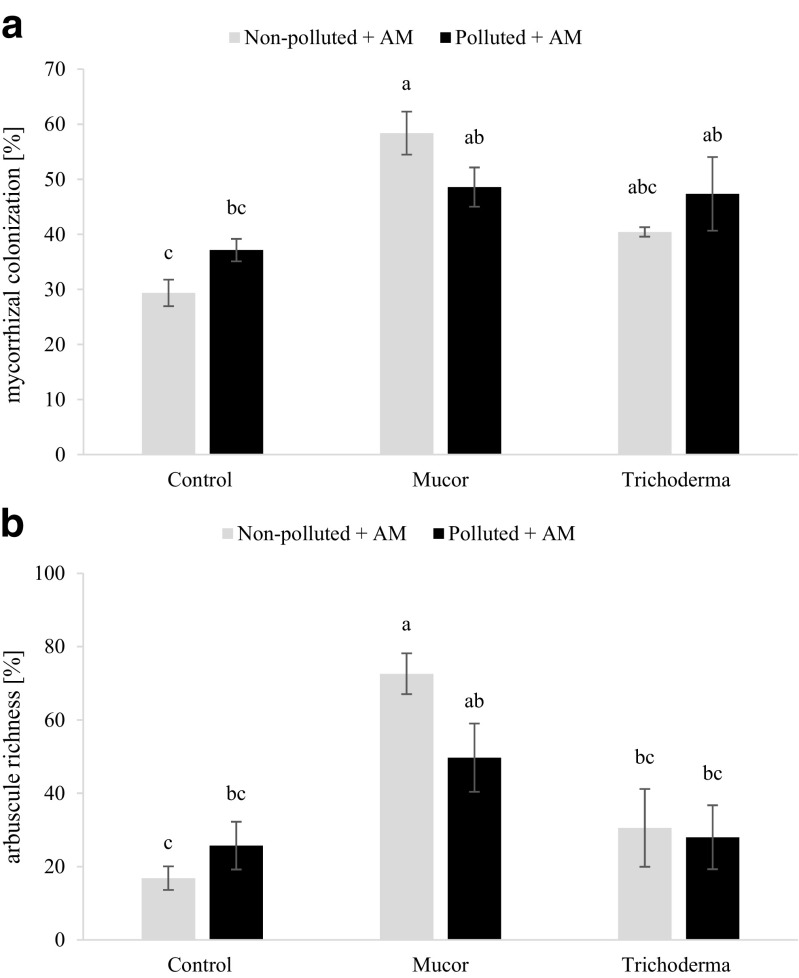
Effect of endophytes *Mucor* sp. and *Trichoderma asperellum* on the absolute mycorrhizal colonization (**a**) and arbuscule abundance (**b**) of plants inoculated with *Rhizoglomus intraradices* and cultivated on non-polluted (NP) and polluted (P) substrates; bars topped by the same letter do not differ significantly at *P* ≤ 0.05 by Tukey’s test; error bars show standard error

The frequency of mycorrhiza (F%) in plant roots was very high and reached nearly 100% in each AM fungus treatment (data not shown). Mycorrhizal colonization intensity (m%) ranged between 29 and 58% depending on the treatment. *Mucor* sp. significantly increased mycorrhizal colonization only in NP substrate (Fig. 4a). *T. asperellum* did not influence mycorrhizal colonization of the roots in either substrate (Fig. 4a). *Mucor* also significantly increased arbuscule abundance (a%) only in plants grown in NP soil (Fig. 4b). In plants cultivated without AM fungus inoculum, mycorrhizal structures were not observed.

### Toxic metal concentration in plant tissues

**Fig. 5:**
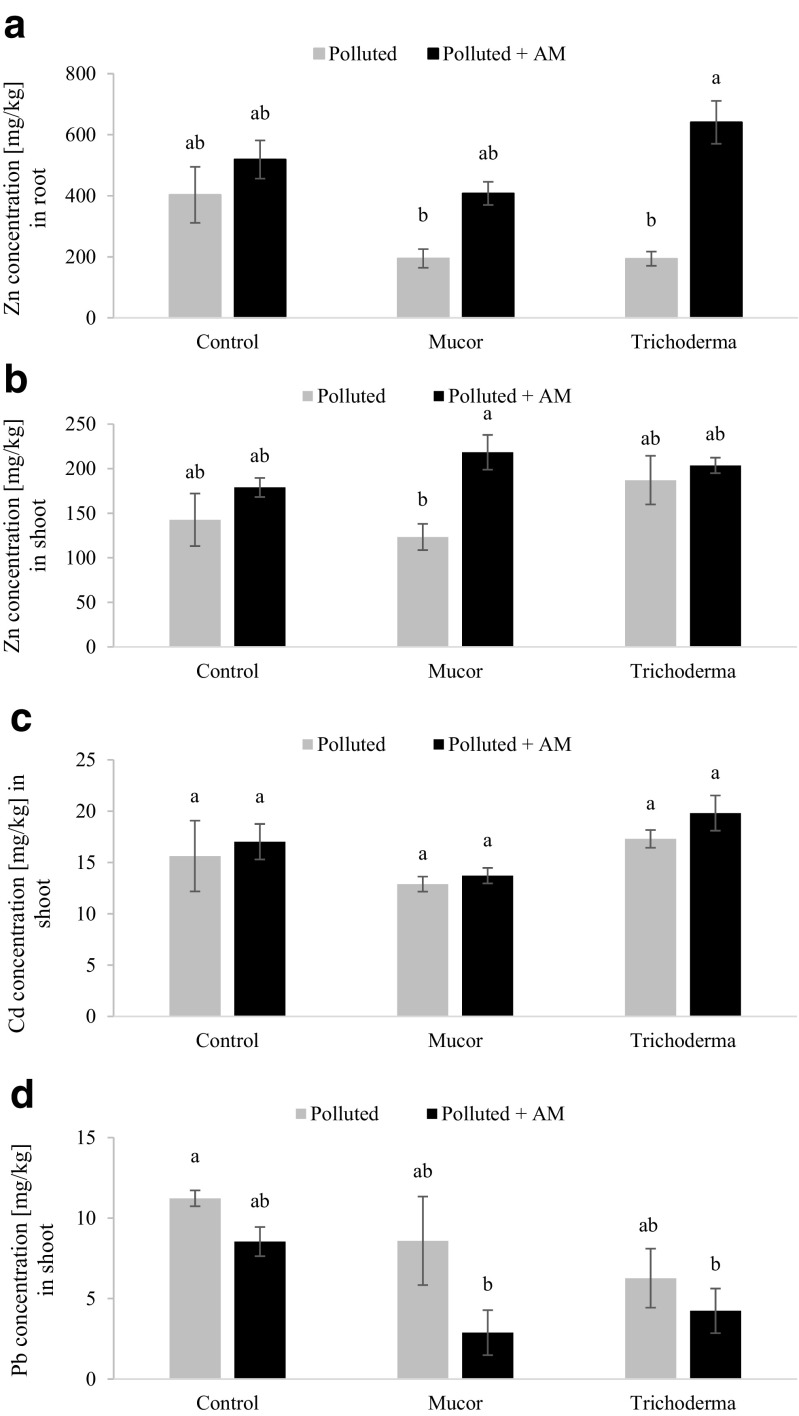
Toxic metal concentration of *Lactuca serriola* inoculated with AM (*Rhizoglomus intraradices*) and endophytic (*Mucor sp*. and *Trichoderma asperellum*) fungi and cultivated on polluted substrate: Zn in root (**a**), Zn in shoot (**b**), Cd in shoot (**c**), Pb in shoot (**d**). Bars topped by the same letter do not differ significantly at *P* ≤ 0.05 by Tukey’s test; error bars show standard error

Zn concentration in non-inoculated *L. serriola* roots reached 403 mg/kg. Single inoculation with AM fungus and with endophytic fungi did not affect it (Fig. 5a). Co-inoculation with *T. asperellum* and AM fungus significantly increased Zn concentration in comparison to single inoculation with *T. asperellum* (3.3-fold; Fig. 5a). Zn concentration in shoots was not affected by single inoculation with AM fungus or endophyte. Co-inoculation with *Mucor* sp. and AM fungus increased this parameter in comparison to single inoculation with endophyte (1.7-fold; Fig. 5b). Zn translocation from root to shoot was the most effective in plants inoculated singly with *T. asperellum* (49%) and *Mucor* (39%). In the case of entirely non-inoculated plants, Zn translocation was 26%.

The concentration of Cd in leaves of AM fungus and endophytic fungi inoculated *L. serriola* ranged from 13 to 20 mg/kg and did not differ from controls (Fig. 5c). Pb concentration in leaves ranged from 3 to 11 mg/kg, depending on the treatment (Fig. 5d).

### Sesquiterpene lactone content

**Fig. 6:**
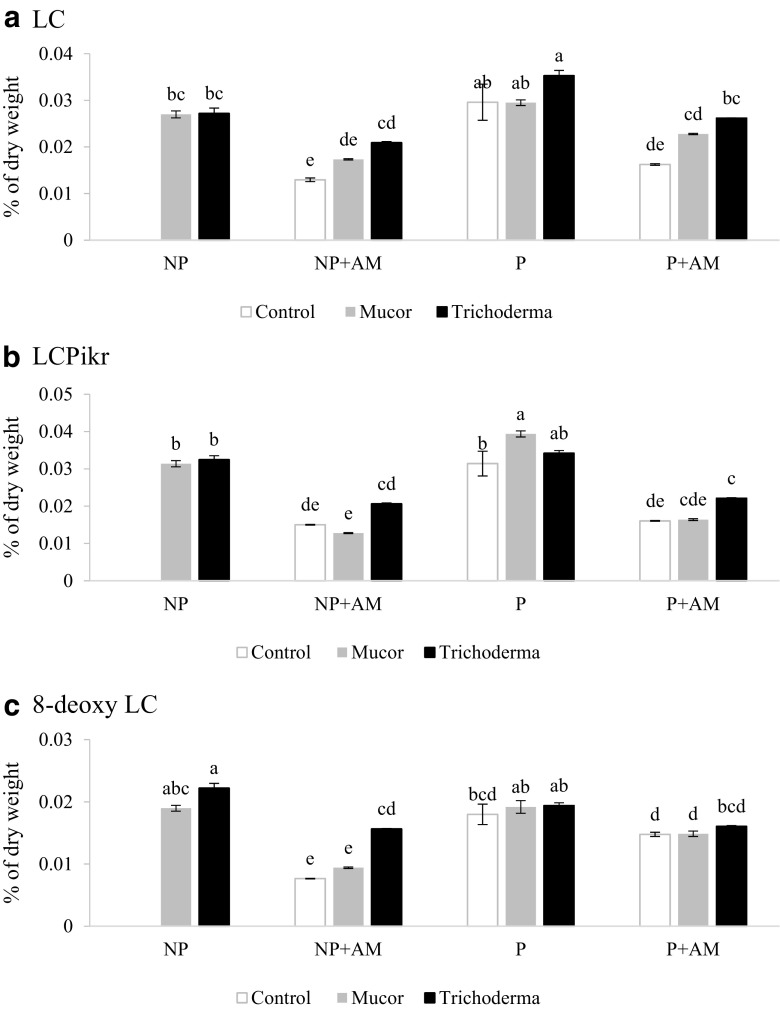
Concentration of sesquiterpene lactones in leaves of *Lactuca serriola* inoculated with AM (*Rhizoglomus intraradices*) and endophytic (*Mucor* sp. and *Trichoderma asperellum*) fungi and cultivated on non-polluted (NP) and polluted (P) substrates:> Lactucin (LC) (**a**), Lactucopicrin (LCPikr) (**b**),8-Deoxylactucin (8-DeoxyLC) (**c**). Bars topped by the same letter do not differ significantly at *P* ≤ 0.05 by Tukey’s test; error bars show standard error

Because of the low dry biomass of the non-inoculated plants in NP substrate, analysis of the secondary metabolites was not possible. In NP substrate, however, co-inoculation of endophyte-inoculated plants with AM fungus decreased the concentration of lactucin (LC; Fig. 6a), lactucopicrin (LCPikr; Fig. 6b), and 8-deoxy lactucopicrin (8-deoxy LC; Fig. 6c) in comparison with the plants inoculated either with *Mucor* or with *T. asperellum* as a sole endophyte.

In P substrate, *Mucor* sp. increased lactucopicrin concentration in leaves (Fig. 6b). Single inoculation with AM fungus significantly decreased LC and LCPikr concentration (Fig. 6a, b). When *Mucor* sp. inoculated plants were co-inoculated with AM fungus, LCPikr and 8-deoxy LC concentrations significantly decreased (Fig. 6b, c). Co-inoculation with AM fungus and *T. asperellum* decreased LC and LCPikr concentration (Fig. 6a, b).

## Discussion

In this study, we have shown that all three considered factors (inoculation with mycorrhizal and endophytic fungi and supplementation of the substrate with TM) had various effects on *L. serriola* plants. The growth response of the plant to the AM fungus was the strongest and was independent of the substrate condition: all AM plants yielded more biomass than their respective controls. Additionally, the concentration of flavonoids was significantly lower, indicating stress attenuation for AM plants. Co-inoculation with endophytic fungi amplified the growth response but differed between fungus species and substrate. The beneficial effects of *Trichoderma* were restricted to NP soil. In the presence of TM, no significant growth difference in *Trichoderma* inoculated plants was found, independently of the presence/absence of the AM fungus, suggesting that these fungi cannot be beneficial to the plant (in terms of growth acceleration) under metal toxicity. *Mucor* sp., on the other hand, exerted its effect only under the presence of TM in combination with the AM fungus (*Mucor* sp. co-inoculated plants yielded more biomass than AM fungus alone plants). This indicates that under metal toxicity, this fungus species can complement the AM fungus. Most importantly, our study shows that the effects of mycorrhizas can be amplified by co-inoculation. Studying the response of plants to toxic metals by including diverse fungi in the experiment reveals potentially important synergism among the fungi.

Endophytic fungi such as those in the genera *Cryptosporiopsis*, *Phialocephala* and *Stagonospora* (Schultz 2006 and references therein) and mycorrhizal fungi share the same niche inside root tissues, and both depend on the same carbon source. This suggests that these two groups of fungi might compete with each other for space and resources. In our experiment, however, mycorrhizal colonization only was affected by *Mucor* sp. in NP substrate. Although similar trends were observed in P substrate, the differences were not statistically significant. These results indicate that co-inoculation with an endophytic fungus, such as *Mucor*, can improve colonisation by an AM fungus, and thus, it could have potential in the production of inoculum. What is important is that no differences were found under the resource-limited conditions of the mine dump substrate. In such an environment, severe nutrient and water limitation may have a negative impact on the symbiosis. Here, we found that the presence of the endophyte does not affect mycorrhizal colonisation and arbuscular richness in the polluted substratum. An interesting complementary investigation would be the behaviour of the fungal endophyte in such conditions. This, however, requires further research.

Benefits imposed upon fungal colonisation of both mycorrhizal and endophytic fungi may be associated with improved photosynthesis efficiency (Ruiz-Lozano et al. 1996; Sheng et al. 2008; Rozpądek et al. 2014, 2015, 2016). Here we show an additive effect of co-inoculation, in terms of plant growth accompanied by improved PSII efficiency. AM fungus inoculation improved the two main photosynthesis indices PI_abs_ and PI_total_ in NP substrate_._ The effect was sustained upon co-inoculation with *Trichoderma* but not under metal toxicity (no growth response), providing further evidence for the role of the abiotic environment in determining the three-way interaction between lettuce, *Trichoderma* and the AM fungus. Interestingly, inoculation with *Trichoderma* without AM fungus had a positive effect on plant growth but did not affect photosynthesis, suggesting that the fungus impact on electron transport efficiency is conferred by the mycorrhizal fungus. PSII efficiency in plants grown on P, in contrast to NP substrate, was not changed upon single mycorrhizal or endophyte inoculation nor co-inoculation, although co-inoculation enhanced the contribution of the light reactions for primary photochemistry in comparison to non-inoculated plants. Previous studies carried out on *Verbascum lychnitis* with a similarly polluted substrate (30 km from presently investigated area, Wężowicz et al. 2015) have shown that the presence of mycorrhiza did not change PSII efficiency; however, AM fungus inoculation was able to improve the photosynthesis rate of plant–endophyte consortium which was negatively affected by inoculation with the single endophytic fungus, *Diaporthe* sp. (Wężowicz et al. 2017). The quantum yield of primary photochemistry and the ability to transfer electrons from PSII to PSI were upregulated by co-inoculation with an AM fungus and the endophytic fungus *Diaporthe* sp. (Wężowicz et al. 2017) similarly to our AM fungus—*Mucor* sp. and AM fungus—*Trichoderma* models in the present study.

According to the literature, inoculation with AMF usually resulted in increasing chlorophyll content in host plants (Abdel-Fattah and Mohamedin 2000; Zuccarini 2007; Vafadar et al. 2014). Baslam et al. (2011, 2013b) reported that the symbiosis of lettuce with AMF increased the amount of chlorophyll in leaves. According to the same authors (Baslam et al. 2013a), however, inoculation also can have the opposite effect. In our study, mycorrhizal plants yielded 3–4-fold higher biomass than their respective non-mycorrhizal controls, but chlorophyll *a* concentration was decreased by 20%. This indicates that photosynthesis was improved because of an upregulation of the efficiency of electron transport within the photosystems and not by increasing the number of functional reaction centres.

Plants in general developed two strategies allowing then to withstand high quantities of TM in the substrate (Baker 1981). The first one is the development of a sophisticated network of intrinsic detoxification mechanisms allowing plants to accumulate TM in high concentrations. The second strategy allows plants to prevent TM uptake (avoidance). Cultivated crop lettuce as well as its relatives, wild lettuce species, are known for their ability to accumulate relatively high amounts of toxic metals (Pb, Cd, As, Zn) in their leaves and roots (Cobb et al. 2000). The contribution of mycorrhizal fungi to toxic metal uptake by the plant is dependent on metal concentration (Leyval et al. 1997). At high metal concentration in soil, mycorrhizas reduced Zn and Cd accumulation, but at low concentration increased Zn and decreased Cd accumulation in lettuce shoots (Schüepp et al. 1987). In the present study, mycorrhizas alone did not affect the accumulation of toxic metals. Inoculation with the endophytic *Mucor* sp. alone decreased the Zn concentration in plant roots. Surprisingly, in contrast, co-inoculation with *Mucor* sp. increased the Zn concentration in roots and shoots. These results indicate that various fungi and their combinations play different, often opposite, roles in influencing a host’s strategy to TM stress. This might be important in controlling plant metal homeostasis in phytoremediation applications. The results presented here confirm observations reported recently (Rozpądek et al. 2018). In *Arabidopsis arenosa*, grown in polluted substrate from the “Bolesław” mine dump, inoculation with *Mucor* sp. affected plant growth and metal homeostasis. Inoculated *A. arenosa* accumulated less Zn and translocated Cd from root to shoots more efficiently than in non-inoculated plants. Additionally, *Mucor* sp. activated root to shoot metal translocation which was accompanied by upregulation of several metal transporter genes (Rozpądek et al. 2018). This indicates the importance of endophytes in adaptation of plants to toxic environments.

Plants synthesize a wide variety of phytochemicals that are required for basic metabolism and are essential for the interaction between the plant and the environment in processes associated with defence and signalling. Plant polyphenols and monophenolics are a group of phytochemicals whose potential for ameliorating environmental stress in plants has been especially well documented (Quideau et al. 2011; Giovannetti et al. 2013; Sbrana et al. 2014). Even mild environmental stresses such as heat shock, chilling and high light intensity induced 2–3-fold phenolic compound concentration increases in cultivated lettuce (Oh et al. 2009). Here, we quantified the abundance of the phenols: caftaric acid, chlorogenic acid, cichoric acid, coniferin, 3,5-dicaffeoylquinic acid, 4,5-dicaffeoylquinic acid, as well some unidentified caffeic acid derivatives and found that their concentrations were decreased by mycorrhizal inoculation (data not shown). Additionally, AM fungus inoculation decreased total flavonoid concentration in leaves, whereas endophytic fungi, *Mucor* and *T. asperellum*, did not affect it. These observations led us to speculate that mycorrhizas decreased TM stress. In this context, the AM fungus seems to be more important for *L. serriola* in decreasing TM-induced plant stress than the endophytes.

Another group of secondary metabolites frequently reported in plants of the *Lactuca* genus is sesquiterpene lactones, particularly 8-deoxylactucin, jacquinelin, crepidiaside B, lactucin, 11β,13-dihydrolactucin, lactucopicrin and lactuside A (Michalska et al. 2009). These lactones are accumulated within laticifers as a constitutive component of latex and have anti-herbivore properties. We detected lactucin, lactucopicrin and 8-deoxylactucin in *L. serriola* leaves. The same sesquiterpene lactones were detected in *Cichorium intybus* leaves, where they provided a significant barrier against herbivory (Rees and Harborne 1985). The concentration of the sesquiterpene lactones analysed in plant leaves was decreased by AM fungus inoculation in plants grown in both soil treatments, but not by fungal endophytes in single inoculation experiments. This implies that the synthesis of these compounds may be downregulated to facilitate colonisation by AMF, but downregulation is not required during the plant-endophyte interaction. This implies variation in mechanisms of AM fungus and endophyte interaction. Co-inoculation with endophytic fungi increased sesquiterpene lactone concentration in some cases (compared to AM fungus inoculated plants) but did not restore it to levels found in plants lacking mycorrhizas, suggesting that co-inoculation with endophytic fungi tended to restore the plant sesquiterpene lactone phenotype. There was no difference in the AM fungus colonisation between single and co-inoculation treatments, so the observed effect did not result in lower AM fungus colonisation caused by the endophyte. In the available literature, the effect of AMF on sesquiterpene lactone production in plants also is not clear. In the case of *Arnica montana*, only a minority of AM fungus inocula investigated was able to increase the concentration of these compounds in shoots, while the rest of the AMF tested did not affect it (Jurkiewicz et al. 2010). In *C. intybus* shoots, mycorrhizas did not affect sesquiterpene lactone production (Rozpądek et al. 2014).

In conclusion, the results presented here indicate that inoculation of *L. serriola* with arbuscular mycorrhizal fungi significantly improved plant biomass in polluted and non-polluted substrates. Additional inoculation with endophytic fungi *Mucor* sp. or *T. asperellum* enhanced this beneficial effect. Co-inoculation of the plants with an AM fungus and *Mucor* also increased Zn concentration in leaves of *Lactuca* and improved mycorrhizal colonisation. Despite that increased host plant costs were potentially caused by maintaining symbiosis with multiple microorganisms, interaction of wild lettuce with both mycorrhizal and endophytic fungi was more beneficial than with a single fungal partner. High tolerance of this plant species to drought supported by mycorrhizas and endophytes improving biomass and affecting toxic metal accumulation shows the potential of application of this model in unfavourable environments.

## Electronic supplementary material

Supplementary figure 1 Variation in the growth of *Lactuca serriola,* nine weeks after inoculation with AM and endophytic fungi (GIF 754 kb)

High resolution image (TIFF 1143 kb)

Supplementary figure 2 Endophytic fungi colonizing *Lactuca serriola*: (a) longitudinal sections of roots colonized by *Mucor* sp., (b) root hairs colonized by *Mucor* sp., (c) longitudinal sections of roots colonized by *Trichoderma asperellum,* (d) *T. asperellum* hyphae developing on the root surface; white arrows indicate fungal hyphae; rh = root hair (GIF 757 kb)

High resolution image (TIFF 1466 kb)

Supplementary Table 1 *P-*values, F-statistics and degrees of freedom (DF) of the three-way analysis of the Chlorophyll a, flavonoids, JIP-test parameters (PI_abs_ – absorbance performance index, PI_total_ – total performace index, φPo/(1-φPo) – contribution of light reactions for primary photochemistry, RC/ABS – fraction of reaction center chlorophyll per chlorophyll of the antennae, Ψo/(1-Ψo) – electron transport beyond primary quinone acceptor and RE/ABS – contribution of the reduction of end equivalents), fresh weight (FW) and dry weight (DW), toxic metal (Zn, Cd Pb) and lactones (lactucin (LC), lactucopicrin (LCPikr), 8-deoxylactucin (8-DeoxyLC )) concentration in *Lactuca serriola* as affected by AM (*Rhizoglomus. intraradices*) and endophytic (*Mucor sp*. and *Trichoderma asperellum*) fungi colonization under toxic metal stress. *P-*values less than 0.05 are considered significant and marked with bold. E – effect of endophyte inoculation; AM – effect of mycorrhizal inoculation; S – effect of substratum; E x AM, S x AM, E x S, E x AM x S – effect of the interaction (DOCX 22 kb)
