# Supplementary figures and images for: Does co-inoculation of Lactuca serriola with endophytic and arbuscular mycorrhizal fungi improve plant growth in a polluted environment?
Source: Mycorrhiza. 2018 Jan 23;28(3):235–46. doi: 10.1007/s00572-018-0819-y (PMC5851704; doi:10.1007/s00572-018-0819-y)

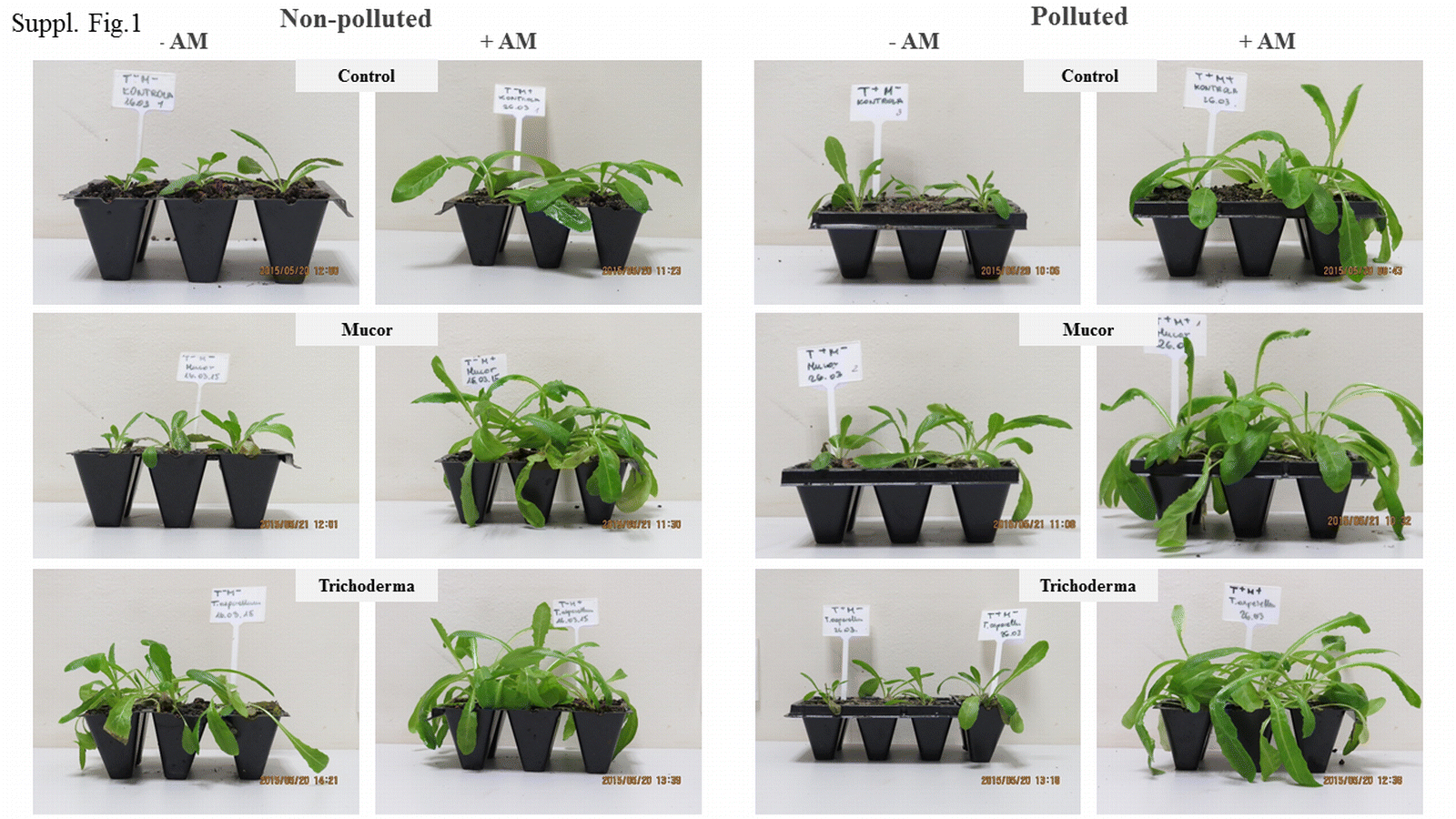

Supplement: Supplementary file 1 — Variation in the growth of Lactuca serriola, nine weeks after inoculation with AM and endophytic fungi (GIF 754 kb) [file 572_2018_819_Fig7_ESM.gif]

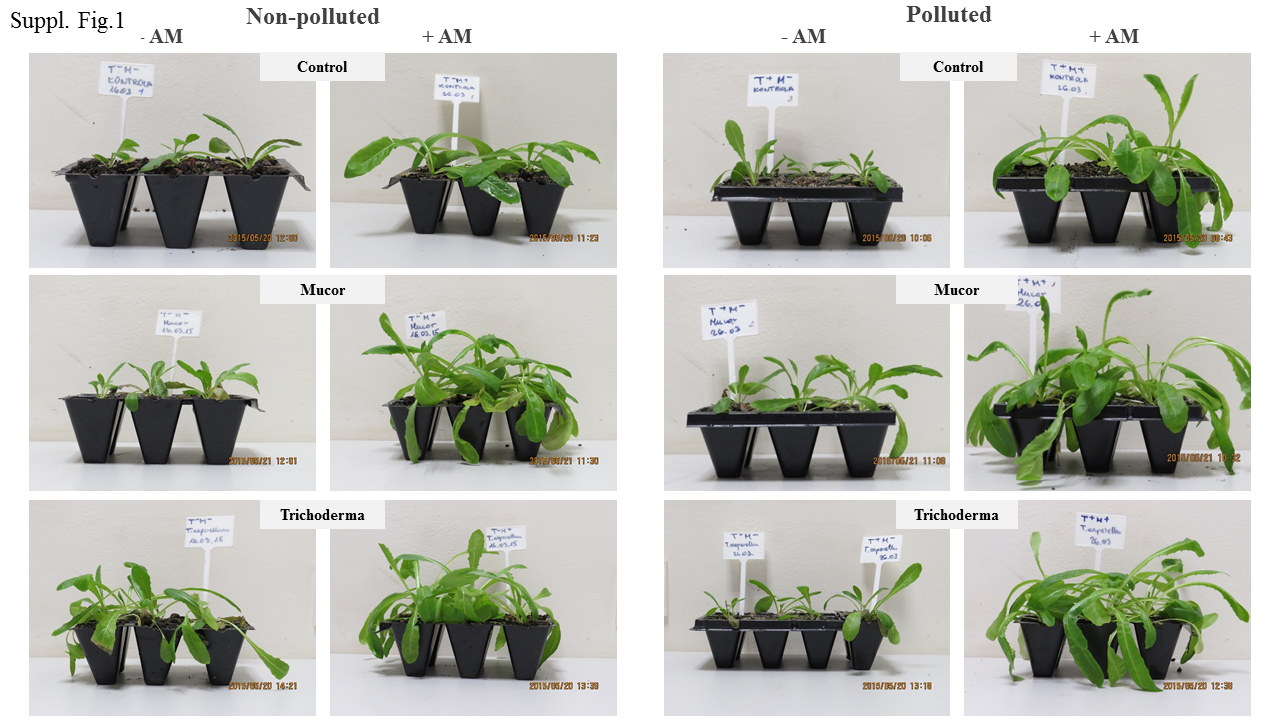

Supplement: Supplementary file 2 — High resolution image (TIFF 1143 kb) [file 572_2018_819_MOESM1_ESM.tif]

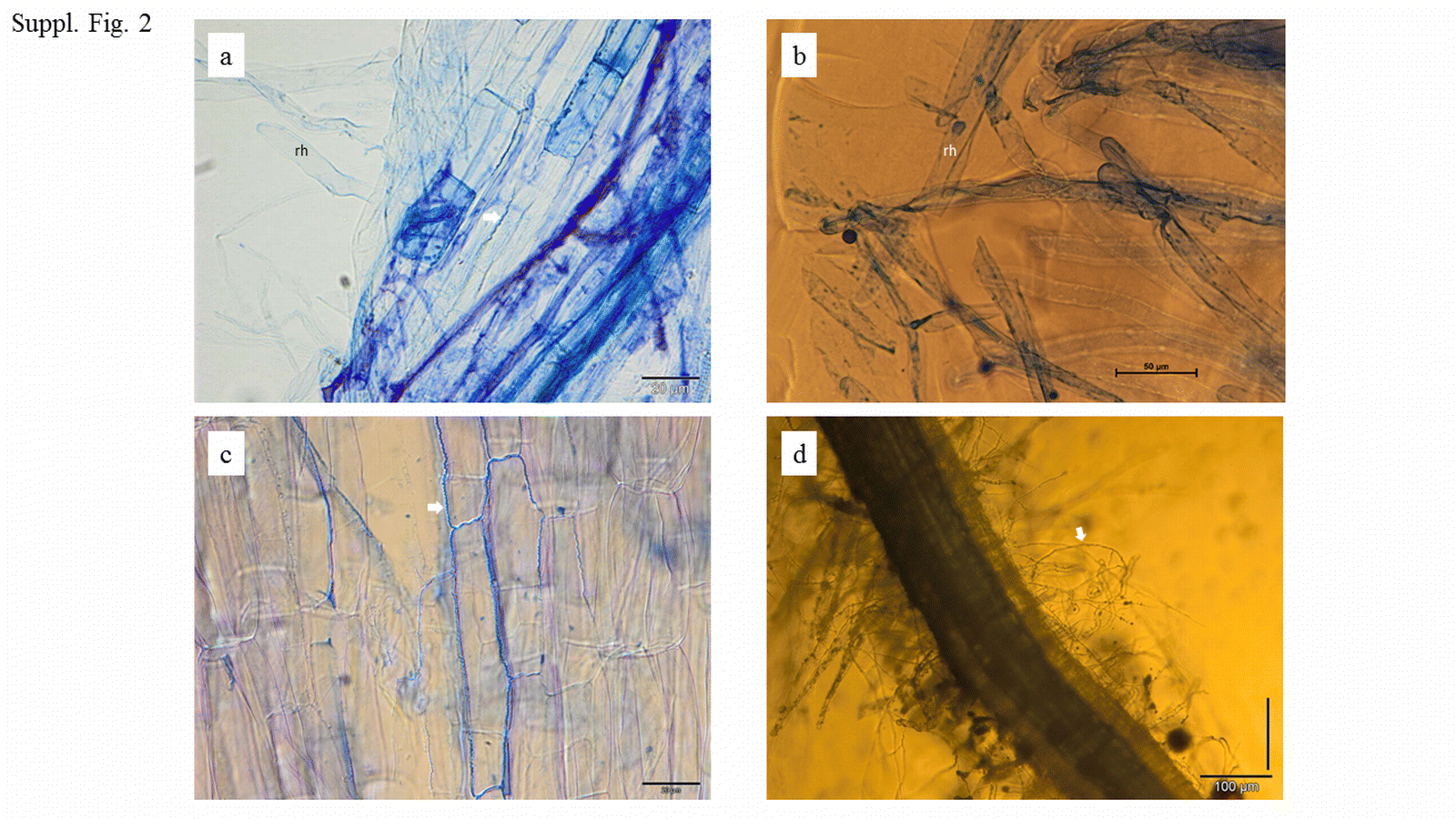

Supplement: Supplementary file 3 — Endophytic fungi colonizing Lactuca serriola: (a) longitudinal sections of roots colonized by Mucor sp., (b) root hairs colonized by Mucor sp., (c) longitudinal sections of roots colonized by Trichoderma asperellum, (d) T. asperellum hyphae developing on the root surface; white arrows indicate fungal hyphae; rh = root hair (GIF 757 kb) [file 572_2018_819_Fig8_ESM.gif]

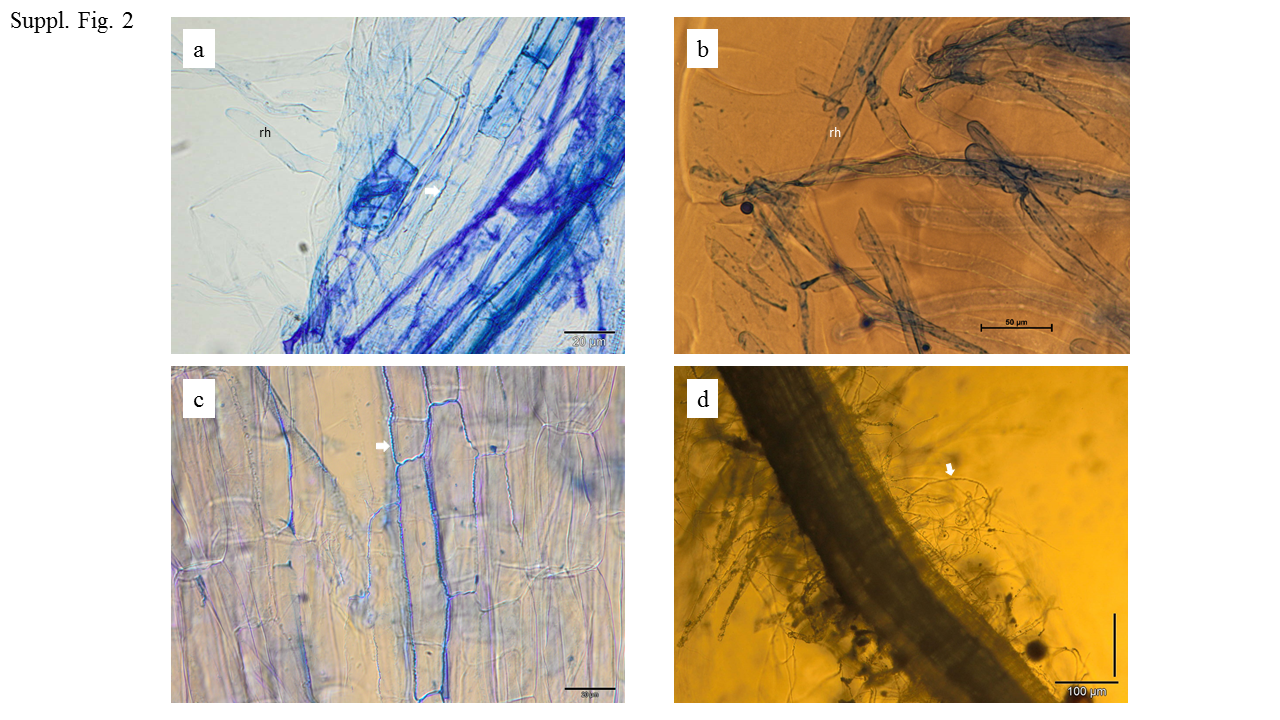

Supplement: Supplementary file 4 — High resolution image (TIFF 1466 kb) [file 572_2018_819_MOESM2_ESM.tif]
