# Supplementary material for: Does co-inoculation of Lactuca serriola with endophytic and arbuscular mycorrhizal fungi improve plant growth in a polluted environment?
Source: Mycorrhiza. 2018 Jan 23;28(3):235–46. doi: 10.1007/s00572-018-0819-y (PMC5851704; doi:10.1007/s00572-018-0819-y)
Supplement: Supplementary file 5 — P-values, F-statistics and degrees of freedom (DF) of the three-way analysis of the Chlorophyll a, flavonoids, JIP-test parameters (PIabs – absorbance performance index, PItotal – total performace index, φPo/(1-φPo) – contribution of light reactions for primary photochemistry, RC/ABS – fraction of reaction center chlorophyll per chlorophyll of the antennae, Ψo/(1-Ψo) – electron transport beyond primary quinone acceptor and RE/ABS – contribution of the reduction of end equivalents), fresh weight (FW) and dry weight (DW), toxic metal (Zn, Cd Pb) and lactones (lactucin (LC), lactucopicrin (LCPikr), 8-deoxylactucin (8-DeoxyLC )) concentration in Lactuca serriola as affected by AM (Rhizoglomus. intraradices) and endophytic (Mucor sp. and Trichoderma asperellum) fungi colonization under toxic metal stress. P-values less than 0.05 are considered significant and marked with bold. E – effect of endophyte inoculation; AM – effect of mycorrhizal inoculation; S – effect of substratum; E x AM, S x AM, E x S, E x AM x S – effect of the interaction (DOCX 22 kb) [file 572_2018_819_MOESM3_ESM.docx]

|  |  | Main factor effect | |  | Significant interaction | | | |
| --- | --- | --- | --- | --- | --- | --- | --- | --- |
|  |  | S | AM | E | S x AM | S x E | AM x E | S x AM x E |
| Chlorophyll a | p-value | 0.661 | **<0.001** | **0.024** | 0.624 | **0.019** | 0.152 | 0.140 |
|  | F-statistic | 6.844 | **25.890** | **3.051** | 7.034 | **1.223** | 0.017 | 4.758 |
|  | DF | 1 | **1** | **2** | 1 | **2** | 2 | 2 |
| Flavonoids | p-value | 0.588 | **<0.001** | 0.206 | 0.966 | **0.049** | 0.202 | 0.129 |
|  | F-statistic | 6.423 | **178.639** | 1.365 | 0.870 | **1.434** | 2.013 | 0.412 |
|  | DF | 1 | **1** | 2 | 1 | **2** | 2 | 2 |
| RC/ABS | p-value | 0.808 | **0.024** | 0.653 | **0.017** | 0.739 | 0.550 | 0.349 |
|  | F-statistic | 0.060 | **5.247** | 0.427 | **5.867** | 0.303 | 0.601 | 1.062 |
|  | DF | 1 | **1** | 2 | **1** | 2 | 2 | 2 |
| ϕPo/(1-ϕPo) | p-value | 0.293 | **<0.001** | 0.665 | **0.001** | 0.982 | 0.762 | 0.857 |
|  | F-statistic | 1.110 | **30.250** | 0.410 | **10.770** | 0.020 | 0.270 | 0.150 |
|  | DF | 1 | **1** | 2 | **1** | 2 | 2 | 2 |
| Ψo/(1−Ψo) | p-value | 0.842 | 0.306 | **<0.001** | **0.001** | 0.770 | 0.648 | 0.316 |
|  | F-statistic | 0.040 | 1.060 | **11.920** | **11.300** | 0.260 | 0.440 | 1.160 |
|  | DF | 1 | 1 | **2** | **1** | 2 | 2 | 2 |
| PI_abs_ | p-value | 0.960 | **0.022** | 0.703 | **0.001** | 0.964 | 0.694 | 0.620 |
|  | F-statistic | 0.000 | **5.430** | 0.350 | **11.600** | 0.040 | 0.370 | 0.480 |
|  | DF | 1 | **1** | 2 | **1** | 2 | 2 | 2 |
| RE/ABS | p-value | 0.748 | 0.187 | 0.053 | **0.001** | 0.645 | 0.691 | 0.533 |
|  | F-statistic | 0.100 | 1.760 | 3.010 | **12.650** | 0.440 | 0.370 | 0.630 |
|  | DF | 1 | 1 | 2 | **1** | 2 | 2 | 2 |
| PI_total_ | p-value | 0.996 | **0.035** | 0.662 | **0.001** | 0.961 | 0.672 | 0.600 |
|  | F-statistic | 0.000 | **4.550** | 0.410 | **11.980** | 0.040 | 0.400 | 0.510 |
|  | DF | 1 | **1** | 2 | **1** | 2 | 2 | 2 |
| Plant FW | p-value | 0.069 | **<0.001** | **0.001** | 0.914 | 0.166 | 0.279 | 0.362 |
|  | F-statistic | 3.600 | **157.000** | **10.100** | 0.000 | 1.900 | 1.300 | 1.100 |
|  | DF | 1 | **1** | **2** | 1 | 2 | 2 | 2 |
| Plant DW | p-value | 0.347 | **<0.001** | 0.930 | 0.744 | 0.847 | 0.443 | 0.270 |
|  | F-statistic | 0.920 | **24.070** | 0.070 | 0.110 | 0.170 | 0.840 | 1.380 |
|  | DF | 1 | **1** | 2 | 1 | 2 | 2 | 2 |
| Zn | p-value | --- | **0.010** | 0.304 | --- | --- | 0.210 | --- |
|  | F-statistic | --- | **7.613** | 1.243 | --- | --- | 1.650 | --- |
|  | DF | --- | **1** | 2 | --- | --- | 2 | --- |
| Cd | p-value | --- | 0.479 | 0.242 | --- | --- | 0.960 | --- |
|  | F-statistic | --- | 0.475 | 1.504 | --- | --- | 0.040 | --- |
|  | DF | --- | 1 | 2 | --- | --- | 2 | --- |
| Pb | p-value | --- | **0.016** | **0.013** | --- | --- | 0.508 | --- |
|  | F-statistic | --- | **6.743** | **5.279** | --- | --- | 0.698 | --- |
|  | DF | --- | **1** | **2** | --- | --- | 2 | --- |
| LC | p-value | --- | --- | **0.002** | --- | 0.156 | 0.797 | 0.135 |
|  | F-statistic | --- | --- | **12.360** | --- | 2.160 | 0.070 | 2.400 |
|  | DF | --- | --- | **1** | --- | 1 | 1 | 1 |
| LCPikr | p-value | --- | --- | **0.007** | --- | **0.016** | **<0.001** | 0.212 |
|  | F-statistic | --- | --- | **8.800** | --- | **6.880** | **30.200** | 1.650 |
|  | DF | --- | --- | **1** | --- | **1** | **1** | 1 |
| 8-deoxy LC | p-value | --- | --- | **<0.001** | --- | **<0.001** | 0.050 | 0.298 |
|  | F-statistic | --- | --- | **32.200** | --- | **17.600** | 4.300 | 1.100 |
|  | DF | --- | --- | **1** | --- | **1** | 1 | 1 |
| ---- - not tested because of insufficient tissue | | | |  |  |  |  |  |
